# Severe Pre-eclampsia Presenting With Breathlessness: A Diagnostic and Management Challenge

**DOI:** 10.7759/cureus.87670

**Published:** 2025-07-10

**Authors:** Aravinda Hariram

**Affiliations:** 1 Obstetrics and Gynaecology, Royal Surrey County Hospital, Guildford, GBR

**Keywords:** breathlessness, hypertension, maternal morbidity, pneumonia, pre-eclampsia, pregnancy, pulmonary edema

## Abstract

Pre-eclampsia is a multisystem disorder that can complicate pregnancy and contribute to significant maternal morbidity and mortality. When complicated by pulmonary edema and pneumonia, which are rare but life-threatening, it presents a diagnostic and management challenge due to overlapping respiratory symptoms and potential delays in identifying concurrent conditions. We report the case of a 28-year-old woman at 35 weeks of gestation who presented with severe breathlessness. She was diagnosed with severe pre-eclampsia complicated by pulmonary edema and concurrent pneumonia. Initial symptoms developed within 24 hours of presentation, and early interventions, including oxygen supplementation, antihypertensive therapy, and antibiotics-were initiated promptly upon admission. Due to rapid clinical deterioration and peri-arrest condition, a timely category 1 cesarean section was performed after efforts towards stabilization to facilitate maternal resuscitation. The patient required supplemental oxygen, intravenous antihypertensives, and close multidisciplinary monitoring. Both maternal and neonatal outcomes were favorable. This case highlights the importance of early recognition and comprehensive management of atypical presentations of pre-eclampsia. In pregnant women with hypertension presenting with breathlessness, a high index of suspicion for pulmonary complications, particularly edema and infection, is essential. Prompt diagnosis and multidisciplinary care are critical to optimizing maternal and fetal outcomes.

## Introduction

Maternal collapse is a rare but potentially fatal obstetric emergency. Maternal collapse is defined as an acute event involving the cardio-respiratory systems and/or central nervous systems, resulting in a reduced or absent conscious level (and potentially cardiac arrest and death), at any stage in pregnancy and up to six weeks after birth [1]. Although infrequent, maternal collapse carries significant risk for both maternal and fetal morbidity and mortality. Prompt recognition and timely, multidisciplinary intervention are essential to optimizing outcomes.

Pre-eclampsia is a hypertensive disorder of pregnancy that affects approximately 2-8% of pregnancies and can have multi-systemic implications [2]. When complicated by pulmonary edema and pneumonia, which are rare but life-threatening, it presents a diagnostic and management challenge due to overlapping respiratory symptoms and potential delays in identifying concurrent conditions. These complications significantly increase the risk of maternal collapse, particularly in previously healthy primigravida who may not be immediately suspected of having severe underlying pathology [2]. In pregnant women with hypertension presenting with breathlessness, a high index of suspicion for pulmonary complications, particularly thrombus and infection, is essential. Prompt diagnosis and multidisciplinary care are critical to optimizing maternal and fetal outcomes.

We report the case of a 28-year-old woman at 35 weeks of gestation who presented with severe breathlessness. She was diagnosed with severe pre-eclampsia complicated by pulmonary edema and concurrent pneumonia. Initial symptoms developed within 24 hours of presentation, and early interventions, including oxygen supplementation, anti hypertensive therapy, and antibiotics, were initiated promptly upon admission. Due to rapid clinical deterioration and peri-arrest condition, a timely category 1 cesarean section was performed after efforts at stabilization to facilitate maternal resuscitation. The patient required supplemental oxygen, intravenous anti hypertensives, and close multidisciplinary monitoring. This case highlights the importance of early recognition and comprehensive management of an atypical presentations of pre-eclampsia.

## Case presentation

A 28-year-old primigravida with a known history of pre-eclampsia, managed with oral labetalol, presented at 35 weeks of gestation with a history of worsening breathlessness. On arrival at the maternity assessment unit, she appeared acutely unwell, with an oxygen saturation of 92% on room air and a blood pressure of 150/100 mmHg, despite adherence to antihypertensive therapy. She was tachypneic and dyspneic at rest. Auscultation revealed normal heart sounds but bilateral basal crepitations.

Given her respiratory compromise and hypertensive disorder, she was urgently transferred to the high-dependency unit (HDU). Initial differential diagnoses included pulmonary embolism, peripartum cardiomyopathy, acute coronary syndrome, and pneumonia, in addition to pulmonary edema. Investigations to evaluate these possibilities were initiated pre-operatively, including ECG, echocardiogram, and a point-of-care ultrasound (POCUS).

Her oxygen saturation declined further despite high-flow oxygen at 15 L/min. Blood pressure remained elevated despite oral labetalol and nifedipine. Arterial blood gas (ABG) revealed type 1 respiratory failure with the following parameters: pO_2_: 6kPa (reference range: 10.5-13.5 kPa), pCO_2_: 3.8 kPa (reference range: 4.7-6.0 kPa), pH: 7.32 (reference range: 7.35-7.45), lactate: 3.6 mmol/L (reference range: 0.5-2.2 mmol/L), indicating hypoxic respiratory failure with mild respiratory alkalosis and raised lactate.

Laboratory investigations showed hemoglobin 9 g/dL (reference range in pregnancy: 11-14 g/dL), platelets 175 × 10^9^/L (reference range: 150-400 × 10^9^/L), and normal liver enzymes. 

Her respiratory status deteriorated rapidly, within 15-20 minutes of admission, with oxygen saturation dropping from 80% to 15% upon lying flat for ECG. She became unresponsive and entered a peri-arrest state, requiring immediate resuscitation. Cardiopulmonary resuscitation (CPR) was initiated for approximately two minutes, during which bag-mask ventilation with 100% oxygen, chest compressions, and one dose of intravenous epinephrine (1 mg) were administered. The return of spontaneous circulation (ROSC) was achieved promptly. No intubation was required at this point.

Given her critical condition, a Category 1 cesarean section under general anesthesia was performed within 30 minutes of presentation, in a semi-recumbent position to support ventilation and allow ongoing resuscitative efforts. Magnesium sulfate was administered at a standard loading dose of 4 g IV over 20 minutes, followed by a maintenance dose of 1 g/hour, in accordance with seizure prophylaxis guidelines. Renal function was monitored to adjust dosing if needed, though no dose modification was required.

Intraoperatively and postoperatively, she received intravenous labetalol, cautious fluid resuscitation, and close fluid balance monitoring. Positive pressure ventilation was continued in the ICU, with ventilator settings optimized to manage both pulmonary edema and infection-related lung injury.

**Figure 1:**
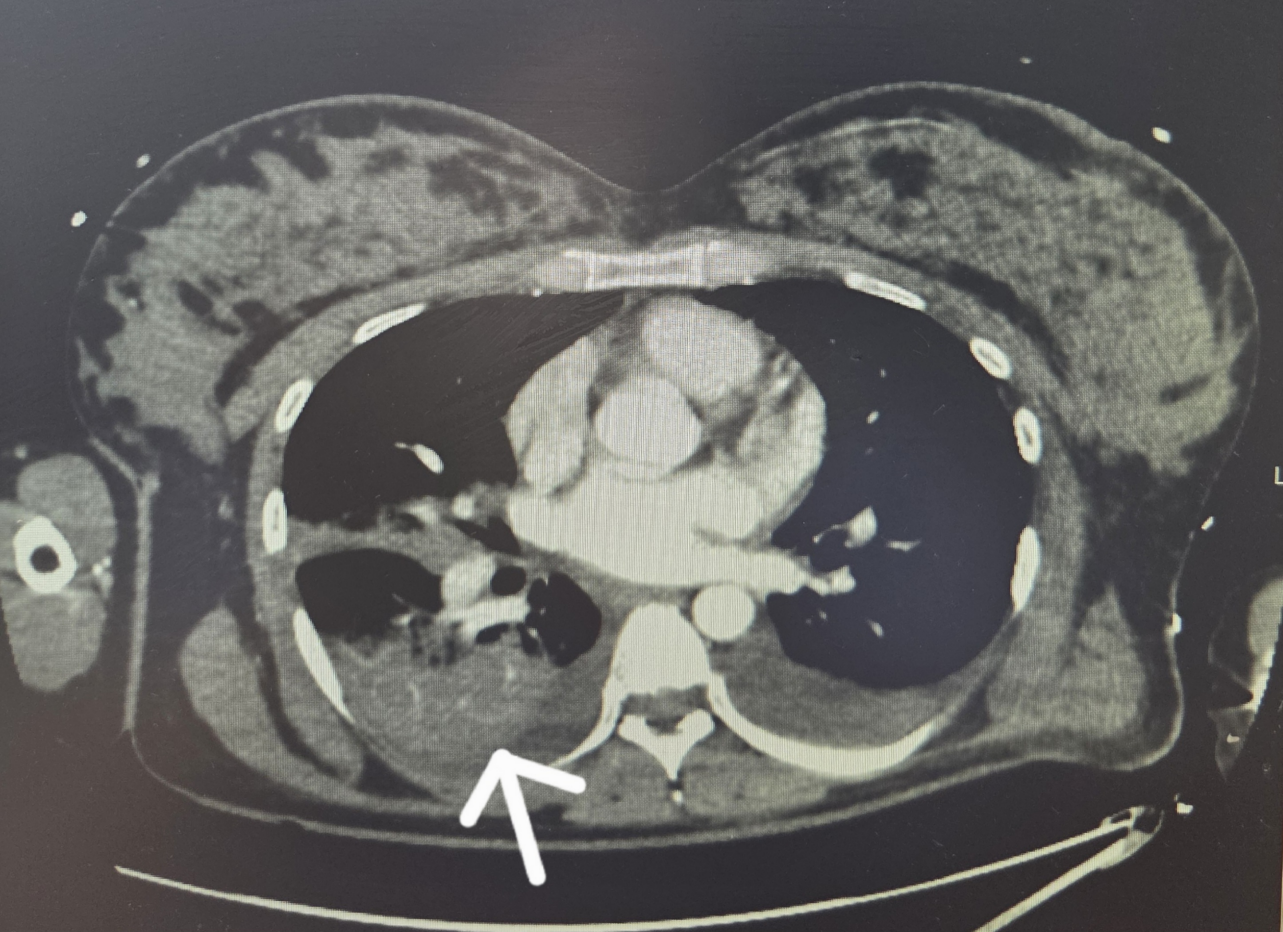
CT thorax with contrast showing dense consolidation within the right lower lobe (arrow)

A high-resolution CT scan postoperatively revealed bilateral pulmonary edema and dense consolidation in the right lower lobe, consistent with pneumonia (Figure 1).

The diagnosis of pneumonia was based on radiological findings and elevated inflammatory markers; no microbial confirmation was obtained, as blood and sputum cultures were negative. She was started empirically on IV co-amoxiclav, considering the likely community-acquired etiology and her pregnancy status. The dual pathology of pulmonary edema and pneumonia required careful management of ventilatory support, strict fluid restriction, and broad-spectrum antibiotics, balancing the needs of both cardiac and infectious etiologies. She was extubated successfully 36 hours postoperatively and transferred out of ICU in a stable condition.

The neonate was delivered in good condition, cried immediately after birth, and had reassuring Apgar scores. The baby was admitted to the Special Care Baby Unit (SCBU) for observation and made an uneventful recovery.

At six weeks postpartum, the maternal chest X-ray and inflammatory markers, including CRP and procalcitonin, had normalized. Her blood pressure remained stable, and antihypertensive therapy was no longer required.

## Discussion

Pre-eclampsia is a pregnancy-specific hypertensive condition that typically presents after 20 weeks of gestation with elevated blood pressure and, in many cases, proteinuria. It is associated with systemic endothelial dysfunction, vasospasm, and increased capillary permeability, which can lead to complications across various organ systems, particularly the cardiovascular and respiratory systems [3,4]. One of the more serious complications is pulmonary edema, which may develop due to a combination of reduced plasma oncotic pressure, increased vascular leakiness, and heightened systemic vascular resistance. Acute pulmonary edema is frequently observed in the postpartum period in patients with pre-eclampsia due to poor fluid management. However, it is not very common antenatally. When such women present with breathlessness, it is essential to consider a wide range of differential diagnoses, including cardiac conditions, pulmonary embolism, and pneumonia [4].

The pneumonia was likely community-acquired, given the absence of prior hospitalization and onset of symptoms before admission, and the CT scan played a central role in decision-making. It showed focal consolidation in the right lower lobe, supporting the diagnosis. Although the blood and sputum cultures were negative, elevated inflammatory markers and radiographic findings met the diagnostic criteria for a clinical diagnosis. She was treated empirically with intravenous co-amoxiclav for seven days, with clinical and biochemical improvement, justifying its role in the dual pathology. Also, hypoxemia progresses more quickly in pregnant individuals compared to those who are not pregnant, making prompt, high-quality airway and breathing management crucial. A higher partial pressure of oxygen is needed to maintain equivalent maternal oxygen saturation levels. This underscores the need for prioritizing maternal oxygenation and ventilation alongside effective chest compressions during resuscitation [5]. Airway management in pregnant patients is inherently more challenging and should be approached with this consideration in mind. As such, pregnancy-specific airway protocols should be followed. For first responders with limited airway management experience, bag-mask ventilation using 100% oxygen remains the quickest and most effective noninvasive method to begin ventilatory support [6].

The clinical presentation of acute breathlessness, hypoxia, and basal crepitations prompted immediate consideration of pulmonary edema, a known complication of pre-eclampsia, but the differential diagnosis was broad. Peripartum cardiomyopathy was considered in light of respiratory compromise and pregnancy status, but echocardiography revealed normal biventricular function and no regional wall motion abnormalities. Pulmonary embolism (PE) was suspected due to acute hypoxia, but CT pulmonary angiography ruled it out. Acute coronary syndrome was excluded based on normal ECG findings and absence of chest pain or troponin elevation [7]. Ultimately, the coexistence of pulmonary edema and pneumonia was confirmed by clinical and radiological findings.

The successful outcome in this case was largely due to the coordinated response of a multidisciplinary team. Obstetricians, anesthesiologists, intensivists, and neonatologists collaborated effectively to ensure timely resuscitation, safe cesarean delivery, and postnatal care. Airway management could also be challenging due to obesity and airway edema, a known risk in pre-eclamptic patients, requiring a second intubation attempt [8]. This did not occur in our situation as we proactively included early senior anesthetic involvement, preoxygenation in an upright position, preparation of difficult airway equipment, and the use of a semi-recumbent position during cesarean section to reduce airway collapse and optimize oxygenation.

The decision to proceed with emergency cesarean delivery likely averted further maternal deterioration by relieving physiological stress, particularly reduced venous return and diaphragmatic splinting. Post-operatively, the patient was managed in the ICU with positive pressure ventilation and targeted antibiotic therapy. Her gradual recovery, including normalization of blood pressure and resolution of pulmonary findings, underscores the importance of early escalation, high-index clinical suspicion for multi-organ involvement, and meticulous peripartum fluid management in patients with severe pre-eclampsia. Globally, hypertensive disorders of pregnancy, particularly pre-eclampsia, remain one of the leading causes of maternal mortality, accounting for approximately 14% of maternal deaths [9]. This highlights the need for further research and development of standardized protocols to guide rapid, multidisciplinary responses in managing rare but severe cardiopulmonary complications of pre-eclampsia, especially in resource-limited settings.

## Conclusions

Antepartum pulmonary edema secondary to a dual pathology, pre-eclampsia and pneumonia, is an exceptionally rare cause of maternal collapse. It constitutes a critical obstetric emergency that poses significant risks to both the mother and fetus. This case highlights the need for the early identification of cardiopulmonary complications, such as acute pulmonary edema, which can quickly progress to life-threatening respiratory failure. Accurate differential diagnosis and timely, multidisciplinary management are essential to optimize outcomes. Successful treatment hinges on close monitoring, prompt escalation of care, and individualized supportive interventions, including appropriate timing of delivery and careful postpartum surveillance.
